# Smoking determines the 10-year (2004–2014) prognosis in patients with Acute Coronary Syndrome: the GREECS observational study

**DOI:** 10.1186/s12971-015-0063-6

**Published:** 2015-11-25

**Authors:** Venetia Notara, Demosthenes B. Panagiotakos, Semina Kouroupi, Ifigenia Stergiouli, Yannis Kogias, Petros Stravopodis, George Papanagnou, Spyros Zombolos, Yannis Mantas, Antonis Antonoulas, Christos Pitsavos, for the GREECS Study Investigators, Greece

**Affiliations:** Department of Nutrition and Dietetics, School of Health Science and Education, Harokopio University, 46 Paleon Polemiston St. 166 74, Glyfada, Athens Greece; First Cardiology Clinic, School of Medicine, University of Athens, Athens, Greece; Cardiology Clinic, General Hospital of Karditsa, Karditsa, Greece; Cardiology Clinic, General Hospital of Zakynthos Island, Zakynthos, Greece; Cardiology Clinic, General Hospital of Lamia, Lamia, Greece; Cardiology Clinic, General Hospital of Kalamata, Kalamata, Greece; Cardiology Clinic, General Hospital of Chalkida, Chalkida, Greece

**Keywords:** Acute Coronary Syndrome, Smoking, Lifestyle habits, Cardiovascular risk factors, Disease clinical burden

## Abstract

**Background:**

Smoking has long been positively associated with the development and progression of coronary heart disease. However, longitudinal cohort studies evaluating smoking habits among cardiac patients as well as the role of socio-demographic factors determining such behaviours are scarce and have been focused on primary care practice. Thus the aim of the present work was to examine the association of active smoking and behaviours and exposure to second-hand smoke, with the 10-year Acute Coronary Syndrome (ACS) prognosis, among cardiovascular patients.

**Methods:**

From October 2003 to September 2004, a sample of six Greek hospitals was selected and almost allconsecutive 2172 ACS patients were enrolled. In 2013–14, the 10-year follow-up was performed in 1918 participants (11 % loss to follow-up). Smoking habits at the time of entry to the study, as well as during the follow-up period were studied using a standard questionnaire.

**Results:**

Patients who had >60 pack-years of smoking had 57.8 % higher ACS mortality and 24.6 % higher risk for any ACS event. Nested model, adjusted only for age and sex, revealed that for every 30 pack-years of smoking increase, the associated ACS risk increased by 13 % (95 % CI 1.03, 1.30, *p* = 0.001). When further adjusted analysis, including several potential confounders, was applied the tested relationship was still significant (95 %CI 1.03, 1.30, *p* = 0.09). Accordingly, the risk for fatal ACS events increased by 8 % for every 30 pack-years of smoking increase (95 % CI 1.03, 1.63, *p* = 0.06). Moreover, 52 % of the patients reported being exposed to secondhand smoke and when further adjustments were made, it was revealed that they had 33 % (95 % CI 1.12, 1.60, *p* = 0.01) higher risk of having recurrent ACS events.

**Conclusions:**

Active smoking and second-hand smoke among cardiac patients still represent a substantial clinical burden. Thus, smoking cessation policies should be incorporated into the long-term therapeutic management.

## Background

The association between cigarette smoking and cardiovascular disease (CVD) has been explored since the early ‘60s, mainly in the Framingham Heart and the Seven Countries studies [1, 2]. Hitherto, scientific evidence is indisputable regarding the inverse effect of smoking, both active and secondhand, on the cardiovascular system [3, 4]. However, it seems that smoking cessation recommendations cannot be successfully achieved and cardiac patients, even shortly after hospital discharge, adopt old habits regarding lifestyle behaviors (i.e. diet, exercise, smoking) [5, 6]. Almost 26 % of ACS active smoking patients, at time of hospital admission, continued smoking after 1 year of follow-up [7]. In the EUROASPIRE II study, 21 % of the patients continued smoking, after 3.5 years of follow-up [8]. Several factors have been suggested as possible risk factors of failure in smoking cessation (relapse) and low adherence to healthy lifestyle habits, such as psychological stress, poor social cognitive profile and lack of social support [9–11]. Considering the above factors, the achievement of smoking cessation can be regarded as a complex and multidimensional process.

In addition, exposure to second-hand smoke has been documented to increase the risk of coronary heart disease by almost 30 % and moreover the risk increases exponentially with years of exposure [12–14]. Moreover, in countries where smoke-free legislation has been strictly implemented, they recorded 17 % declines in hospital admissions for ACS events [15]. It has been documented that smoke-free homes discourage smoking initiation alongside with the encouragement of smoking cessation [16].

Despite the large body of unequivocal evidence regarding the role of both active smoking and second-hand smoke in the involvement of atherosclerotic process, the failure in smoking cessation among ACS patients remains quite high and poses a growing disease burden. Therefore, the aim of the present work, and under the context of the GREECS (GREEk acute Coronary Syndrome) multi-center, longitudinal study [17], was to investigate the association between active smoking and exposure to second-hand smoke and the 10-year risk for fatal or non-fatal ACS events, among patients who had had an acute cardiac event.

## Methods

### Sampling procedure at baseline examination 2003–2004

GREECS is a prospective, observational study that was established in 2003. The main goal of the study was to evaluate the annual incidence of ACS, as well as the role of various CVD risk factors on the development and prognosis of ACS. From October 2003 to September 2004, *n* = 2172 consecutive patients with discharge diagnosis of ACS (i.e., acute myocardial infarction (AMI) or unstable angina (UA)) that were hospitalized in the cardiology clinics or the emergency units of 6 major General Hospitals in Greece (i.e., Hippokration hospital in Athens and the general prefectural hospitals in Lamia, Karditsa, Halkida, Kalamata and Zakynthos island) were enrolled into the study (participation rate varied from 80 to 95 %). The hospitals were selected in order to represent populations with various socio-economic, cultural and regional characteristics. Of the enrolled patients, *n* = 1649 (76 %) were men (65 ± 13 years) and *n* = 523 (24 %) were women (62 ± 11 years) (*p* for age and gender differences <0.001). With the exception of Athens, where there are several other hospitals, all the other hospitals cover the whole population of the aforementioned regions, including urban and rural areas. At entry, as well as during hospitalization biomarkers suggesting cardiac injury and AMI were measured. Moreover a 12-lead electrocardiogram (ECG) was performed and clinical symptoms were evaluated in all patients, by a cardiologist. AMI and UA were defined following the up-to-date definitions [18, 19]. Medical information was retrieved through hospital records.

### Investigated measurements at baseline examination

The baseline examination included a variety of patients’ clinical, biochemical, socio-demographic and lifestyle characteristics. Particularly, socio-demographic and lifestyle characteristics included: age, sex, physical activity, diet and smoking, years at school, financial and marital status and psychological evaluation. In particular, as regards smoking, patients were asked whether they were current, former or never smokers. Current smokers were defined as those who smoked at least 1 cigarette/day or have attempted to quit smoking during the past 12 months, while the rest who smoked at some time were defined as past or former smokers. The rest of the patients were defined as never or occasional smokers [20]. Questions about years of smoking exposure, type of cigarettes smoked (i.e., light, heavy), number of cigarettes/day, smoking at work or/and home place, were asked; for the former smokers information about years of smoking cessation was also recorded. Patients were divided into four quartiles (statistically) according to the distribution of the packs smoked per year: (a) 1^st^ quartile (0 pack/years), (b) 2^nd^ quartile (<30 pack/years), (c) 3^rd^ quartile (30–60 pack/years) and (d) 4^th^ quartile (>60 pack/years). The certain classification provided a better distribution of the sample resulting in balanced subgroups. Special attention was also given to the baseline exposure of second-hand smoke (in years), for at least 30 min per day, to cigarette smoke, at home, workplace, as well as in indoor recreational environments. As regards other major characteristics, financial status was classified – according to the Greek tax cut-offs - as: “low” (<9000€), “moderate” (<18,000€), “good” (<48,000€) and “very good” (>48,000€). Dietary habits were evaluated using a validated food frequency questionnaire and the level of adherence to the Mediterranean dietary pattern was assessed using the MedDietScore (range 0–55) [21]. Higher values of this diet score indicate greater adherence to the Mediterranean diet. Physical activity was evaluated through a self-reported questionnaire provided by the American College of Sports Medicine [22] and it was defined as any engagement in activities of at least 3 times/week and for at least 30 min. As regards medical history, it was retrieved during the physical examination and through the patient’s medical records and included the detailed assessment of hypertension, hypercholesterolemia, diabetes and any previous CVD event (i.e., prior to the baseline), as well as the pharmaceutical treatment and management of these conditions. Body mass index (BMI) was calculated as weight (in Kg) divided by height (in m) squared. Obesity was defined as BMI > 29.9 kg/m^2^.

Further details about the aims, measurements and baseline procedures of the GREECS study may be found elsewhere [17]

### 10-year follow-up evaluation

During 2013–2014, the 10-year follow-up of the patients was performed by the study’s investigators. Information from *n* = 1918 of the initially enrolled patients was retrieved; the remaining *n* = 254 patients were lost after the 1st year of follow-up and considered as censored in the statistical analysis; no vital status information at 10-year was available for these patients (i.e., loss to follow-up around 11 %). Vital status and development of ACS was evaluated using WHO-ICD-9 coding (as it was also performed in the 30-day, 6-month and 1-year follow-up that has been reported in previous publications) [17]. All patients were interviewed by using a standard questionnaire. Smoking during the 10-year follow-up period was also assessed; for current smokers, number of cigarettes/day and years of smoking were asked, while for the former smokers information about the year of smoking cessation was recorded. Moreover, exposure to secondhand smoke was also obtained, following the same methodology described above. Regarding patients who died within the decade and in order to have an accurate death diagnosis, relevant information was retrieved from the medical records, or local mortality registries.

No differences were observed between those participated in the 10-year follow-up and those lost in follow-up, in all baseline clinical and lifestyle factors (all *p*’s >0.50).

### Endpoints at follow-up

The endpoints studied in the 10-year follow-up were recurrent fatal or non-fatal ACS events. In particular, the development of a new AMI, angina pectoris, other identified forms of ischemia (WHO-ICD coding 410–414.9, 427.2, 427.6), heart failure of different types and chronic arrhythmias (WHO-ICD coding 400.0–404.9, 427.0–427.5, 427.9), were recorded by the physicians of the study.

### Bioethics

The study was approved by the Medical Research Ethics Committee of the participating Institutions and was carried out in accordance with the Declaration of Helsinki (1989) of the World Medical Association. All patients were informed about the aims and procedures of the study and signed an informed consent.

### Statistical analysis

Continuous variables are presented as mean values ± standard deviation, while categorical variables are presented as absolute and relative (%) frequencies. Associations between normally distributed continuous variables (i.e., MedDietScore, body mass index and age) and groups of the patients per smoking quartile were evaluated by the analysis of variance (ANOVA), after controlling for equality of variances (homoscedacity). Due to multiple comparisons the Bonferroni rule was applied to correct for the inflation of Type - I error. Years of school variable that was abnormally distributed was tested through Kruskal-Wallis. Associations between categorical variables (i.e., sex, physical activity, financial status, hypertension, hypercholesterolemia, diabetes mellitus, family history of CVD) were tested by the use of the chi-squared test. Survival curves according to quartiles of pack-years of smoking were calculated and log-rank test was implemented to evaluate median follow-up differences between quartiles. In order to control residual confounding, which may exist between smoking and ACS incidence, nested models were estimated. Thus, the association between patients’ smoking (i.e., smoking status estimated with pack-years and smoking cessation after the baseline ACS or continue, within the decade) and the dependent variable (i.e., 10-year ACS fatal/non fatal events), after controlling for the above mentioned potential confounders, was evaluated by the use of nested Cox proportional hazard models. Proportionality of the hazards was graphically tested by plotting the log (-log(survival)) vs. the log- of survival time. First order interactions between age, sex, medical history and smoking were also evaluated for potential stratifying analyses. Appropriate tests for goodness-of-fit (i.e., deviance and Pearson’s residuals) were applied in order to evaluate the robustness of the models’ estimates rather than create prediction models. Results are presented as hazard ratios (HR) and their corresponding 95 % confidence intervals (95 % CI). All statistical calculations were performed with the SPSS version 21 software (IBM Hellas Inc, Athens, Greece).

## Results

### Baseline characteristics by smoking among ACS patients

**Figure 1:**
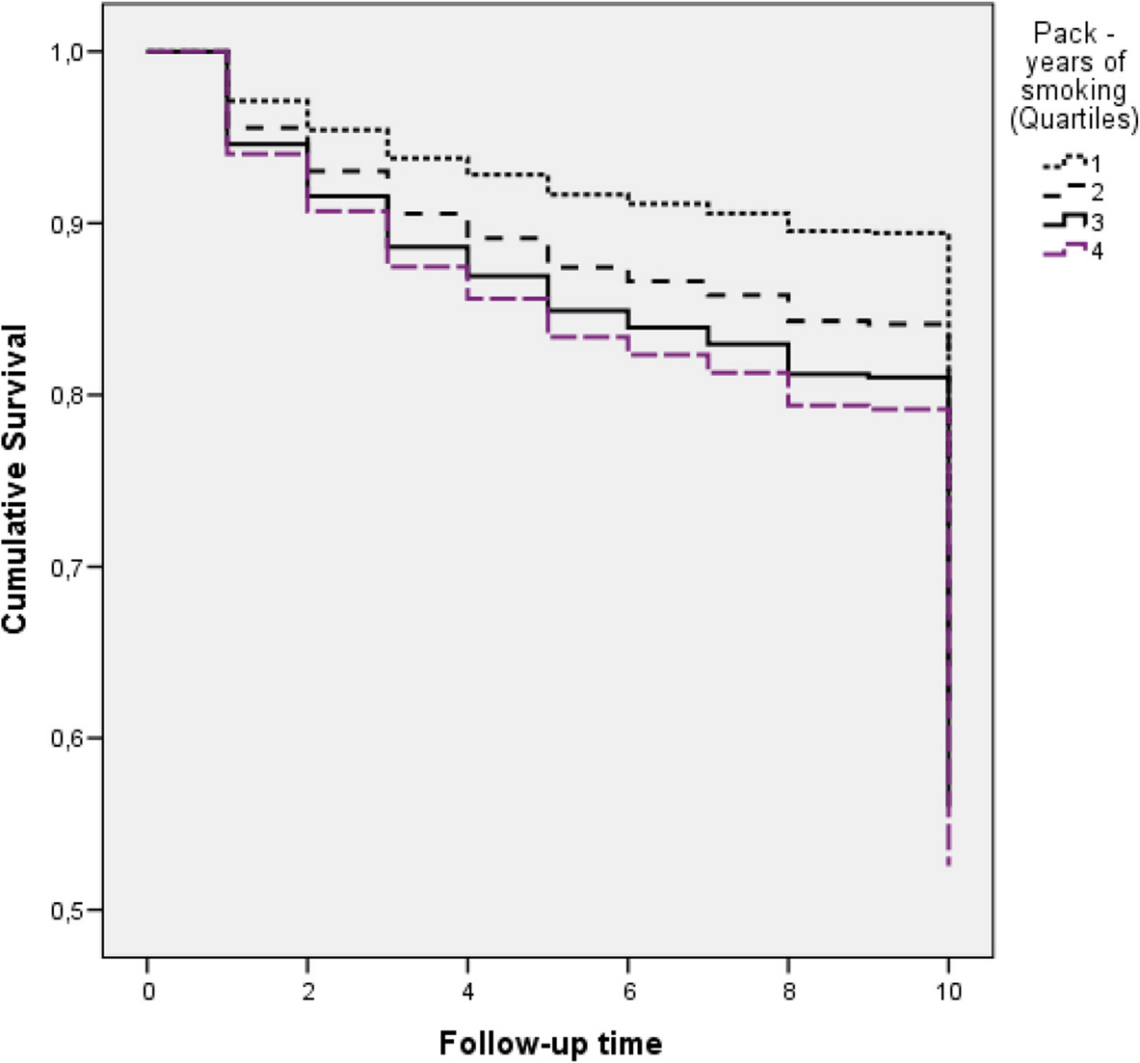
10-year survival curves for ACS^*^ development, according to the quartiles of pack-years of smoking of the *n* = 2172 patients (log-rank *p*-values between 4th, 3rd, and 2nd vs. 1st quartile <0.01, *p*-value between 4th vs. 3rd = 0.78). *ACS event: the development of a new acute myocardial infarction, angina pectoris, other identified forms of ischemia, heart failure of different types and chronic arrhythmias during the 10 years follow-up time

Of the enrolled patients, 34 % reported never smokers, 33 % reported active smokers and 33 % reported that they have quit smoking for an average of 35 ± 39 pack-years (median 30, 1st, 3rd quartile, 0 and 60, respectively), at baseline examination. The vast majority of smokers preferred smoking cigarettes (94 %) and the rest tobacco or pipe. Of the non-smokers, 48 % reported exposed to secondhand smoke at workplace or home, at baseline examination. As it can be seen in Figure 1, patients in the 3rd and 4th upper quartiles of pack-years of smoking had lower survival as compared with those in the lower quartiles (*p* log-rank < 0.01); no difference was observed between 3rd vs. 4th quartile (*p* = 0.78). Moreover, in Table 1 baseline factors that may act as confounders for the evaluation of smoking on 10-year ACS incidence are presented. The analysis showed that patients who were active smokers at the time of first admission (baseline) and were in the upper quartile of pack-years of smoking (i.e., >60) were more likely to be younger age, mainly men, to report alcohol and coffee consumption and to have a family history of CVD. Moreover, patients in the lower quartile of pack-years of smoking were more likely to have low/moderate income, to be less educated, to report history of hypertension and diabetes to be less physically active and to follow a prudent diet close to the Mediterranean pattern.

**Table 1:** Baseline demographic, lifestyle and clinical characteristics of the GREECS study participants, by quartiles of pack-years of smoking (*n* = 2172)

	Quartiles of pack-years				
*Baseline factors*	1^st^	2^nd^	3^rd^	4^th^	*p*
	0	<30	30-60	>60	
	(*n* = 732)	(*n* = 451)	(*n* = 551)	(*n* = 438)	
Age (years),*mean*(*SD*)	72.0(11.4)	63.0(13.1)	62.7(13.0)	64.0(11.8)	<0.001†
Men, %	43	88	95	93	<0.001
Years of school, *mean*(*SD*)	6.4(4.1)	8.7(4.4)	8.2(4.2)	8.3(4.3)	<0.001††
Low/moderate financial status, %	72	62	69	65	0.01
MedDietScore (0–55), *mean*(*SD*)	28.6(5.4)	28.6(5.7)	28.4(5.5)	27.7(6.0)	0.02
Physical activity, %	31	46	41	42	<0.001
Obesity, %	27.5	27.4	27.7	27.7	0.53
Alcohol consumption%	11	32	31	31	<0.001
Cups of coffee/day	0.67	0.86	1.06	1.29	<0.001
History of hypertension, yes %	65	48	45	51	<0.001
History of diabetes, yes %	36	33	27	29	0.01
History of hypercholesterolemia, yes %	44	47	45	49	0.45
Family history of CVD, yes %	33	37	37	42	0.01
History of CVD (prior to baseline event), yes %	60	56	59	58	0.55
*10*-*year follow*-*up data*					
ACS fatal events, %	12.1	16.0^*^	15.2^*^	19.1^*^	0.01^┴^
ACS events overall, %	36.1	33.0	41.0^*^	45.0^*^	0.001^┴^

*p* for trend between groups using: log-rank test (┴), Analysis of Variance (†) or non-parametric Kruskal-Wallis (††). ^*^ *p* < 0.05 for the comparisons between 2^nd^, 3^rd^, 4^th^ vs. 1^st^ quartile of pack-years (reference category), after correcting the probability of the inflation of type I error because of multiple comparisons, using the Bonferroni rule (but, not accounting for age differences)

### Fatal or non-fatal ACS events during the 10-year (2004–2014) of follow-up, by smoking among ACS patients

The median ACS event free follow-up time was 8.3 years for patients in the 1^st^ quartile, 8.2 years for patients in the 2^nd^ quartile, 8.1 years for patients in the 3^rd^ quartile and 7.5 years for patients in the 4^th^ of pack-years (*p* = 0.001). Patients in the 4^th^ quartile of pack-years of smoking had 57.8 % higher ACS mortality and 24.6 % higher risk for any ACS event (Table 1).

In order to control residual confounding, nested models were applied (Table 2). Age- and sex- only adjusted model revealed that for every 30 pack-years of smoking increase the associated ACS risk increased by 13 % (*model 1*). Similarly, when, BMI, MedDietScore and physical activity level, were included in the model as potential confounders, the relationship between smoking and the risk for ACS recurrent events remained significant (Table 2, *model 2*). Additionally, when baseline co-morbidities (i.e., history of hypertension, hypercholesterolemia, diabetes and family history of CVD) were also added in the analysis (Table 2, *model 3*), the tested relationship was still significant. No significant interactions between baseline smoking and age, sex on the 10-year outcome were observed (all *p*’s >0.50).

**Table 2:** Results from nested Cox proportional hazards models regarding the association between pack-years of smoking and 10-year ACS fatal or non-fatal event among cardiac patients (*n* = 2172)

Models for any ACS event	Hazard Ratio	95 % CI	*p*	Model adjusted for:
*Model 1*: per 30 cigarette pack/years	1.13	1.03,1.30	0.001	*Age*, *gender*
*Model 2*: per 30 cigarette pack/years	1.09	1.03,1.30	0.01	*Age*, *gender*, *obesity*, *MedDietScore*, *physical activity*
*Model 3*: per 30 cigarette pack/years	1.06	1.03,1.30	0.09	*Model 2 plus history of hypertension*, *hypercholesterolemia, diabetes and family history of CVD*
Model for fatal ACS events: per 30 cigarette pack/years	1.08	1.03, 1.63	0.06	*Factors used in Model 3*

Cox regression models were applied with the 10-year ACS event as dependent variable and potential confounders gradually adjusted in the models as follows: Model 1: age, gender; Model 2: age, gender, Body Mass Index, MedDietScore, physical activity; Model 3: age, gender, Body Mass Index, MedDietScore, physical activity, history of hypertension, hypercholesterolemia and diabetes mellitus, family history of CVD; Cox regression model was also applied with the 10-year ACS mortality as dependent variable and the following confounders as adjusting factors: age, gender, Body Mass Index, MedDietScore, physical activity, history of hypertension, hypercholesterolemia and diabetes mellitus, family history of CVD

Further analysis was applied to evaluate the association between exposure to secondhand smoke and 10-year occurrence of ACS fatal or non-fatal events. 52 % of the non-smoker patients who had an ACS event during the follow-up, reported that they were exposed to secondhand smoke vs. 46 % of patients who did not have any event (*p* = 0.01). No significant association was observed when the analysis was focused only of fatal ACS events (*p* = 0.45). When further adjustments were made for age, gender, BMI, smoking status, MedDietScore, physical activity, history of hypertension, hypercholesterolemia, diabetes and family history of CVD, it was revealed that patients who reported exposure to secondhand cigarette smoke, at workplace, at home or other places had 33 % higher risk of having a fatal or non-fatal ACS event and 27 % higher risk for a fatal event (Table 3). When the analysis was focused on the source of exposure, no significant results were observed (probably because of the lack of sufficient data).

**Table 3:** Results from nested Cox proportional hazards models regarding the association between exposure to secondhand cigarette smoke and 10-year ACS fatal or non-fatal events, among cardiac patients (*n* = 2172)

*Models for any ACS event*	Hazard Ratio	95 % CI	*p*
Exposed vs. non-exposed	1.33	1.12, 1.60	0.01
Exposed at workplace vs. non-exposed	0.96	0.78, 1.20	0.69
Exposed at home vs. non-exposed	1.13	0.89, 1.43	0.30
*Models for fatal ACS event*			
Exposed vs. non-exposed	1.27	1.01, 1.60	0.05
Exposed at workplace vs. non-exposed	0.87	0.66, 1.14	0.31
Exposed at home vs. non-exposed	1.21	0.99, 1.70	0.25

All models were adjusted for age, gender, BMI, smoking status, MedDietScore, physical activity, history of hypertension, hypercholesterolemia, diabetes and family history of CVD

## Discussion

The present study is one of the very few cohort studies that have investigated active smoking as well as exposure to second-hand smoke, within the 10-year of follow-up, among ACS patients. It was documented that patients who were current smokers and reported smoking for almost 60 pack-years at baseline examination were more likely to be men, of younger age, to have higher alcohol and coffee consumption and have a family history of CVD, as compared with patients in the lower quartile of pack-years of smoking. It was of interest that non-smokers, at baseline examination, were less educated, were in the low/moderate financial group, were less physically active and followed a diet close to the Mediterranean pattern; however, all these associations may be attributed, at least partially, to baseline age differences between smoking groups. Nevertheless, as regards financial status it has been documented that financial crises lead to reduction in smoking prevalence due to reduced affordability of cigarettes [23, 24]. Even though, the vast majority of data support the relationship between educational level and smoking prevalence, our findings are in accordance with the results from nine European countries examining the trends of smoking habits by education group. They observed that smoking declines were higher among the least educated participants, probably attributed to smoking cessation policies such as, free or subsidized access to smoking cessation therapies, telephone help lines, bans on tobacco advertisements, pricing policies [25].

It was also found that patients who, at baseline examination, reported more than 60 pack-years of smoking had 57.8 % higher risk of fatal ACS event and 24.6 % higher risk for any ACS event during the decade, as compared with patients who reported non-smokers; this effect was confirmed even when further adjustments were made. Similarly, even when patients smoked fewer pack-years (i.e. <30 and 30–60 pack-years) the risk remained significantly higher as compared with patients who reported non-smokers. Multivariable analysis, after adjusting for age, sex, as well as BMI, MedDietScore, physical activity level and clinical characteristics (i.e., history of hypertension, hypercholesterolemia, diabetes and family history of CVD), revealed that for every 30 pack-years of smoking increase, the risk for ACS recurrent events was significantly high. Smoking still constitutes a major risk factor for ACS. The Updated Report 2014 of the American Heart Association documented that, regardless the declining rates of smoking since 1998, almost 20.5 % of men and 16 % of women remain current smokers. Regarding health care costs, in 2010 ACS and stroke accounted for 15 % of total health costs, while indirect costs are estimated to increase by 58 % in 2030 [26]. However, previous studies have suggested the “smoker’s paradox” among post-myocardial infarction (MI) patients, in a way that smokers may have a better disease prognosis compared with non-smokers [27, 28]. Nevertheless, the certain phenomenon is likely to be attributed to differences in patients’ socio-demographic characteristics, in different medical treatment plans and mainly to the fact that it was observed only in short-term outcomes [29].

Even though, quit smoking was not analysed in this work - due to some methodological limitations -, it is well documented in the scientific literature that smoking cessation among cardiac patients contributes substantially to the reduction in CVD death rate by almost 40 % [25, 30]. Accordingly, a systematic review of cohort studies resulted in a significant reduction in ACS mortality rate as well as in rehospitalisation for MI, among cardiac patients [31]. However, regardless of the scientific evidence about the benefits of quitting smoking on ACS prognosis, there are equally strong indications that cardiac patients fail to discontinue smoking even immediately after hospital discharge [32, 33].

As regards second-hand smoke, it was revealed that exposure at home or in indoor recreational activities (i.e., pubs, bars, restaurants, etc) was positively associated with the 10-year fatal or non-fatal ACS events, while an inverse association was observed with exposure to tobacco smoke at workplace. Α possible explanations to the aforementioned result could be that most of the patients were retired and had no additional risk deriving from their exposure at work. A meta-analysis study documented that in public locations where comprehensive smoke-free legislation is implemented, the reduction in hospitalizations for ACS reached almost 14 % compared to 8 % in locations with partial smoke-free legislation [34]. Second-hand smoke remains one of the main CVD risk factors, in the same level like active cigarette smoking [35]. In the United States, almost 33.951 of cigarette smoking–related CVD deaths are attributable to secondhand smoke, each year [36]. The possible mechanism through which secondhand smoke is closely related to increased risk of ACS morbidity and mortality is the same to that of active smoking i.e. acceleration of atherosclerosis due to damage of the endothelial lining as well as increased concentration of inflammatory markers [37, 38]. Finally, recent studies have indicated the harmful effects on CVD risk not only of second-hand smoke at home (from spouses/cohabitants) but also the residual tobacco smoke toxic substances long after a cigarette is extinguished (“third-hand smoke” hazards), advocating a rigorous enforcement of smoke-free home policies [39–41].

Several trials have provided results on the effectiveness of cessation counselling and rehabilitation programs based on both behavioural and medication therapy (i.e. Nicotine Replacement Treatment (NRT), bupropion and varenicline) [42–44]. However, the nature of such interventions should be tailored-made according to the disease severity and the patients’ co-morbidities [45].

### Limitations

The present study has some limitations. Recall bias may exist, especially regarding the evaluation of exposure to secondhand smoke. Additionally, social desirability may influence patients’ answers. Smoking status was assessed through self-reporting statement, which might under- or over-estimate the actual behaviour, but this type of data collection is the frequently applied in observational studies [46, 47]. Information regarding the exact date of quitting was not assessed due to the uncertainty of the information provided; thus, it may be hypothesized that some of the patients may have quitted smoking after the new ACS event, and therefore they could be considered as quitters in the present analysis. Smoking among patients was measured at the baseline examination, as well as at the end of the 10-year follow-up therefore they may be prone to life-course changes during the decade and, consequently influence the robustness of the findings. Another limitation could be the fact that the reasons leading patients to quit or continue smoking were not explored since the main purpose of the study was to investigate the association between active smoking and exposure to second-hand smoke and the 10-year risk for fatal or non-fatal ACS events.

## Conclusion

The present study highlighted a crucial issue about current smoking behaviours among cardiac patients, with both active smoking and secondhand smoke playing a detrimental role in the 10-year ACS incidence and mortality. Smoking cessation appeared to reduce the risk of recurrent ACS events by almost half, within the decade. Respectively, exposure to secondhand smoke represented a substantial risk factor in the disease prognosis, with 3 out of 10 patients exhibiting ACS recurrent events (fatal or non-fatal). Intervention strategies regarding smoking cessation and reduced exposure to environmental smoke should be targeted towards cardiac patients in order to further eliminate the disease burden.

## Abbreviations

ACS: Acute Coronary Syndrome
AMI: Acute Myocardial Infarction
BMI: Body mass index
CVD: Cardiovascular disease
ECG: Electrocardiogram
GREECS: GREEk acute Coronary Syndrome
HR: Hazard ratio
UA: Unstable Angina
WHO ICD-9: World Health Organization, International Classification of Diseases Ninth Revision
